# Atopic dermatitis in Taiwanese children: The laboratory values that correlate best to the SCORAD index are total IgE and positive Cheddar cheese IgE

**DOI:** 10.1097/MD.0000000000021255

**Published:** 2020-07-24

**Authors:** Ho-Chang Kuo, Chi-Hsiang Chu, Yu-Jih Su, Chih-Hung Lee

**Affiliations:** a Department of Pediatrics and Kawasaki Disease Center, Kaohsiung Chang Gung Memorial Hospital and Chang Gung University College of Medicine, Kaohsiung; b Clinical Trial Center; c Department of Internal Medicine; d Department of Dermatology, Kaohsiung Chang Gung Memorial Hospital, 83301, Taiwan.

**Keywords:** atopic dermatitis, cheese, immunoglobulin E, SCORAD

## Abstract

Atopic dermatitis (AD) is a common chronic relapsing inflammatory skin disease associated with a personal or family history of atopic diseases. Determining the objective severity scoring of AD index (SCORAD) and total immunoglobulin E (IgE) to help to stage the severity (lesions extent and intensity of the lesions and then the itch and sleep disturbance they may cause) of AD in children.

In this study, we adopted the SCORAD index, which consists of severity, area, and sleep disturbance, to evaluate the AD status of children up to 18 years old. We examined the blood levels of total serum IgE, white blood cell count/differential count (WBC/DC), eosinophil counts (EC), eosinophil cationic protein (ECP) and specific IgE.

A total of 208 children with AD were enrolled in this study. Serum IgE values and a number of specific IgE that are positive significantly different SCORAD index through simple linear regression; however, after multiple linear regression, only IgE values (95% CI: 0.001–0.004, *P* < .001), total WBC count (95% CI: 0.112–1.736, *P* = .026), EC (95% CI: 0.045–6.706, *P* = .047), and specific IgE to Cheddar cheese (95% CI: 1.814–16.731, *P* = .015) remain different. After applying the Phi coefficient, we found that specific IgE to tuna (*r* = 0.632), codfish (*r* = 0.613), and clam (*r* = 0.613) each had a moderate correlation with specific IgE to Cheddar cheese. The 6 most common allergens were found to be mite (D. Farinae: 65.9%), mite (D. Pterony: 64.9%), house dust (47.6%), cockroach mix (37.0%), shrimp (30.8%), and crab (22.6%). Covariates of SCORAD index, severity, area, and sleep disturbance differed.

In this study, we found that total IgE values, specific IgE values, WBC, EC, and specific IgE to Cheddar cheese have significant correlations with SCORAD index in AD of Taiwanese children.

## 1 Introduction

The incidence of allergic diseases (food allergy, asthma, and allergic rhinitis), and atopic disease (atopic dermatitis [AD]) has increased globally.^[1]^ Considerable variations among populations with similar racial/ethnic backgrounds but different environmental exposures indicate that environmental factors contribute significantly to these increase. Atopic dermatitis is a common chronic relapsing inflammatory skin disease that is typically associated with a family history of atopic diseases.^[2]^ AD often announces the presence of other atopic diseases, so studying the early events of the “atopic march” can be helpful with regard to investigating and treating atopic diseases. According to Taiwan's national insurance database survey between 1999 and 2003 for preschool children, around 20% of children have AD and from other report that 15% of AD children have previously had KD history.^[3,4]^

Atopic diseases include a group of allergic diseases defined by the organs they target. The pathogenesis of these diseases seems to involve both significant genetic influences and interactions with environmental allergens. These complicated interactions play vital roles in the disease's onset, severity, and progression. Recent findings in genetic analysis, such as genome wide association studies (GWAS) and new generation sequencing (NGS), have suggested several candidate pathogenetic genes for consideration. For example, filaggrin mutations are observed in approximately 50% of patients with AD. Many immunological regulatory genes (ex: *ORAI1*) have been found to be associated with atopic diseases.^[5]^ In fact, aberrant adaptive immune responses, such as skewed T cell polarization with predominant Th2 responses, are frequently associated with such disease. Eosinophil cationic protein (ECP), elevated blood total immunoglobulin E (IgE), and multiple allergen sensitizations are also regularly present in patients suffering from those diseases. Recently, feeding type of increased protein-hydrolyzed formula (HF) feeding population in Taiwan, as described in our previous report having impact on decreasing food sensitization but not on the development of AD.^[6]^

The assessment of AD patients for general disease severity was using The Severity Scoring of Atopic Dermatitis Index (SCORAD),^[7,8]^ which combines objective (extent and intensity of lesions) and subjective (daytime pruritus and sleep loss) criteria.^[9]^ Serum levels of IgE,^[10,11]^ interleukin (IL)-16,^[12]^ ECP,^[11,12]^ and total eosinphil counts (EC)^[8,10]^ have been reported to correlate with SCORAD index in children with AD and can service as marker for monitoring disease activity. Based on the literature review, few studies have focused on allergens and SCORAD in childhood AD. Therefore, we analyzed clinical laboratory data, including ECP, IgE, complete blood count/differential count (CBC/DC) and allergens with childhood AD via the SCORAD index system in order to provide clinicians with other tools to monitor the severity of AD.

## 2 Methods

### 2.1 Patients studied and SCORAD index

For this study, we recruited children (under the age of 18 years old) who fulfilled the AD criteria (AD is defined as a chronic relapsing pruritic skin rash with dry skin, erythema, or scaling, as well as skin creases, and characteristic areas^[13,14]^) and were treated at the Pediatrics Department of Chang Gung Children's Hospital at Kaohsiung upon obtaining written informed consent from their parents or guardians. AD patients under treatment with systemic steroid or immunosuppressant were excluded for study. This study was approved by the Chang Gung Memorial Hospital's Institutional Review Board (IRB No.: 102-4104A3, 98-3674B). CBC/DC data were collected from central laboratory of hospital for analysis. We assess the severity of AD in these subjects by the SCORAD index. The same trained physician assistant has scored all the children. The higher the SCORAD index, the higher the severity of the AD.

### 2.2 Total serum IgE and specific IgE's

We adopted the Pharmacia CAP system (Pharmacia & Upjohn Diagnostic AB, Uppsala, Sweden) to determine levels of IgE and ECP concentration.^[15]^ Specific allergens were tested using the MAST-CLA system (OPTIGEN; Hitachi Chemical Diagnostics, Inc., Mountain View, CA), which uses an in vitro diagnostic test to simultaneously determine the specific IgE of 36 different allergens. The results were categorized as class 0 to 4 using the MAST Optigen luminometer. Any class ≥1 was considered positive.^[16]^

### 2.3 Data analysis

Data are presented as mean and standard deviation (or median and interquartile range) for continuous variables and as percentage for categorical variables. We applied Pearson correlation and Spearman's rank–order correlation to measure the correlation between 2 random variables. Phi coefficient was adopted to measure the association between 2 nominal variables. We further used partial correlation to analyze the association between 2 random variables by adjusting for the effect of a set of controlling random variables. We adopted a linear regression model to identify the major factors related to the outcomes. A *P* value less than .05 was considered statistically significant. All statistical analyses were performed using IBM SPSS statistical software for Windows version 22.0 (Armonk, NY: IBM Corp).

## 3 Results

### 3.1 Demographic data

We enrolled a total of 208 patients with AD in this study during 2015 to 2017. The SCORAD index (area, sleep disturbance, and severity), demographic data, IgE values, ECP, body weight, gender, age, and positive rate of specific IgE are shown in Table 1. SCORAD index was observed without significance in AD patients between different age groups (*P* = .081), but are significant in relation to the severity of lesions score (*P* = .006) and the area of lesions score(*P* = .008). The age distribution in each group is shown in Table 1 specify the different age groups, <2, 2 to 6 and >6 years of age. A higher level of total IgE (161 ± 255 vs 830 ± 1199 vs 1851 ± 2918 kU/L, *P* < .001, in different age groups respectively), but no difference was found in ECP level (*P* = .61) or eosinophil percentage (*P* = .618). The 6 most common allergens were Mite Farinae (D.f.: 65.9%), Mite Pterony (D.p.: 64.9%), house dust (47.6%), cockroach mix (37.0%), shrimp (30.8%), and crab (22.6%). The 3 least common allergens were Ragweed Mix 1 (1.4%), Timothy Grass (1.9%), and Bermuda Grass (2.4%), all of which have pollen. Positive allergens with pollen or mold were less common in children with AD in Taiwan. The allergen data of the different groups are shown in Tables 2 and 3 including food and environmental allergens.

**Table 1:**
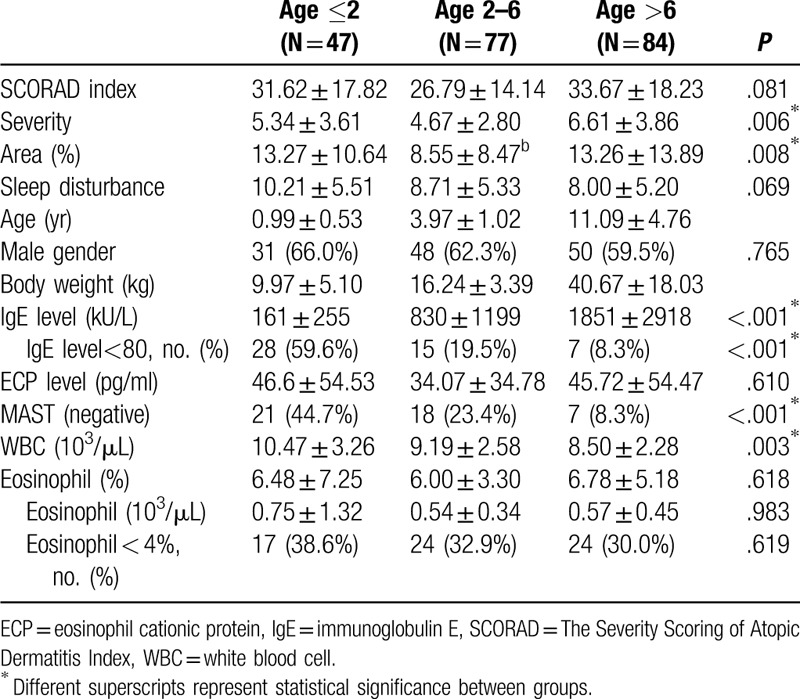
Demographic, laboratory data, and SCORAD index.

**Table 2:**
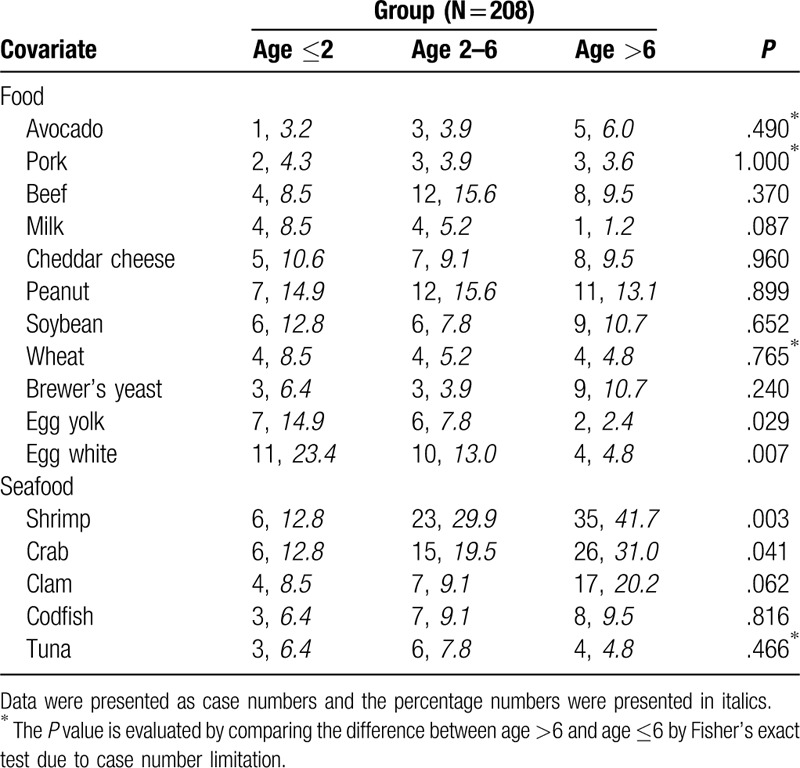
Food allergen data of different age groups.

**Table 3:**
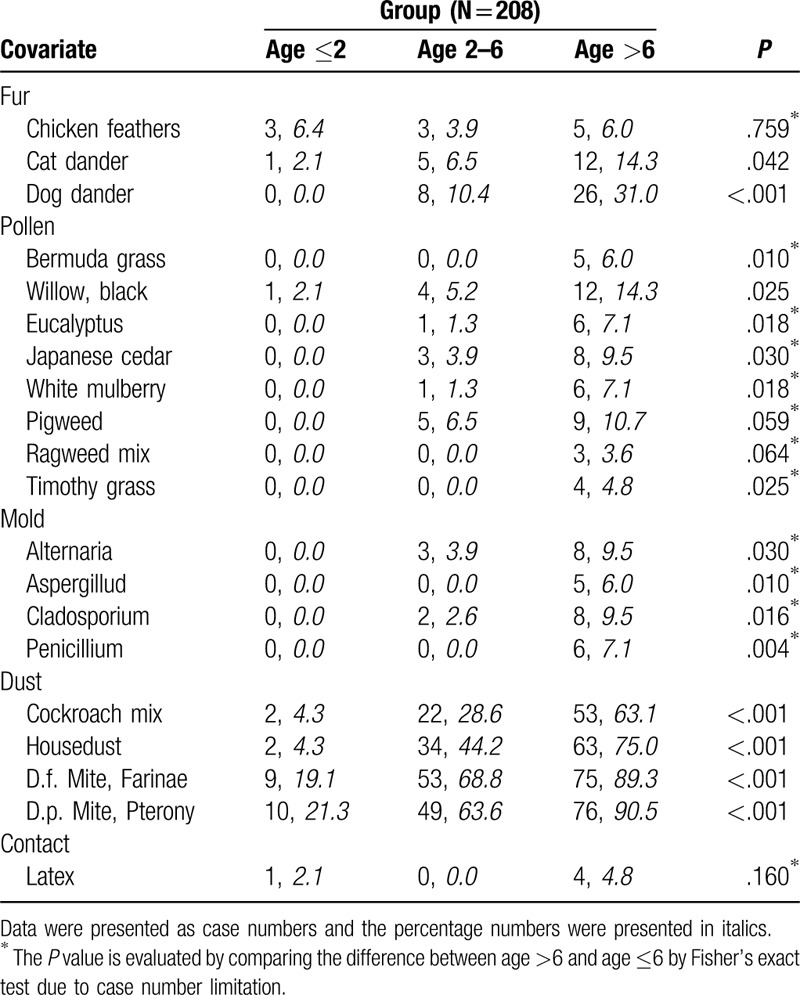
Environmental allergen data of different age groups.

### 3.2 Total serum IgE association with SCORAD index by age group

The correlation coefficient of IgE value was positive with SCORAD index (*P* < .001) (Tables 4–6). After adopting a cut-off age of 6 years old (school age), we found that SCORAD index still demonstrated a positive association with IgE in both age groups (*P* < .05). In the group of patients under 6 years old (N = 124), IgE levels demonstrated a positive relationship with age (*P* < .001) and body weight (*P* < .001), but this is not observed for ECP values (Table 5). In the group of patients older than 6 years old, IgE are positively associated with ECP values (*P* < .001) but not with age or body weight (N = 84, Table 6).

**Table 4:**
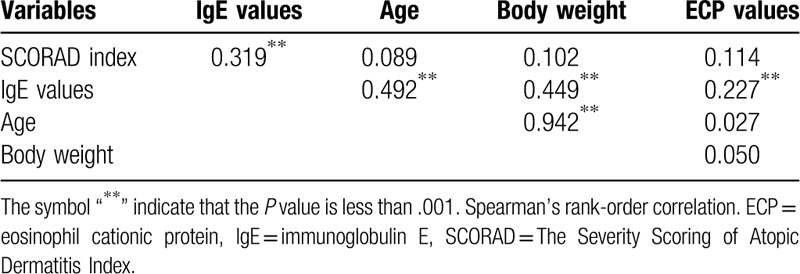
Correlations between variables (N = 208).

**Table 5:**
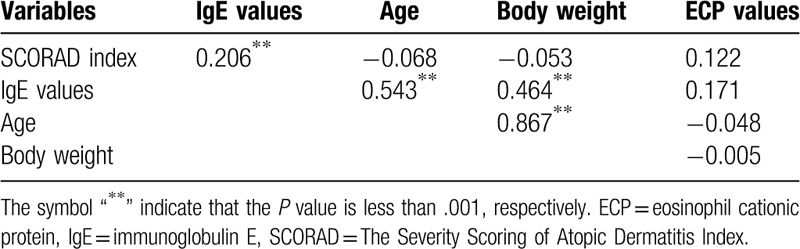
Correlations between variables (age ≤6, N = 124).

**Table 6:**
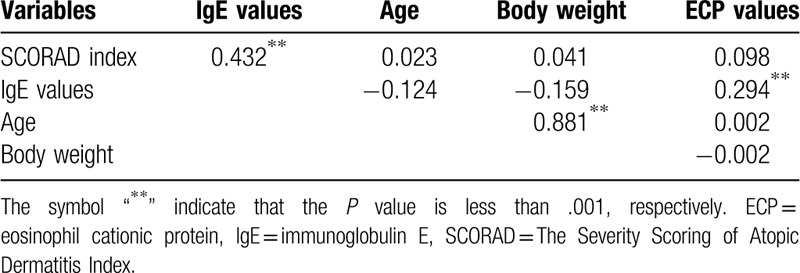
Correlations between variables (age >6, N = 84).

### 3.3 Partial correlation analysis of total serum IgE and SCORAD index

Regarding the under 6 years old group, Tables 7 and 8 show correlations between the following 2 pairs: SCORAD index and IgE level and ECP level and IgE level, pursuant to partial correlation analysis controlling for age and body weight. Nevertheless, IgE levels do not correlate with body weight when controlling for age, while we observed a positive association with age when controlling for body weight (*P* < .001) with partial correlation analysis. There were no significant difference in SCORAD index, severity, and sleep disturbance (*P*> .05), but lower distribution area (%, *P* = .007) when comparing children with only AD to those with multiple allergic diseases. Those who were food sensitized had higher SCORAD index, severity, area, and sleep disturbance (all *P* < .05) when compared to those not food sensitized.

**Table 7:**
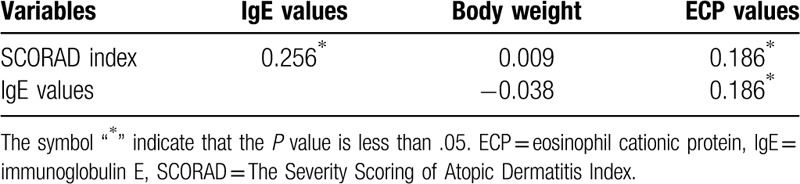
Partial correlations between variables when controlling for age (age ≤6).

**Table 8:**
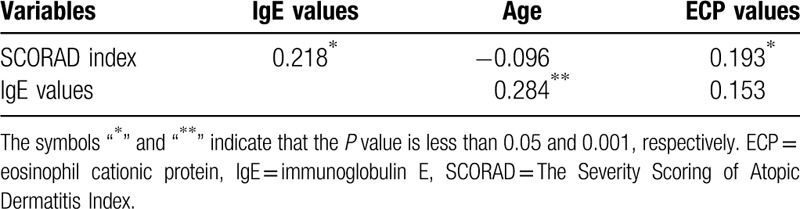
Partial correlations between variables when controlling for body weight (age ≤6).

### 3.4 SCORAD index and Cheddar cheese allergen

To model the relationship between SCORAD index and covariates, we adopted a simple linear regression to select a set of potential covariates and then adjusted multiple linear regressions to determine which covariates from the aforementioned potential set were relevant. In Table 9, IgE level and Cheddar cheese are the important covariates that influence the SCORAD index. Each additional unit of IgE level caused a change of 0.002 in SCORAD index (95% CI: 0.001–0.004, *P* < .001). If a patient is allergic to Cheddar cheese, the AD score is 8.591 (95% CI: 1.415–16.487, *P* = .015) higher than a non-allergic person's index. Each additional unit (10^3^/μL) of WBC count and eosinophil count results in a change of 0.924 (95% CI (0.112–1.736, *P* = .026) and 3.375 (95% CI: 0.045–6.706, *P* = .047) of the SCORAD index, respectively.

**Table 9:**
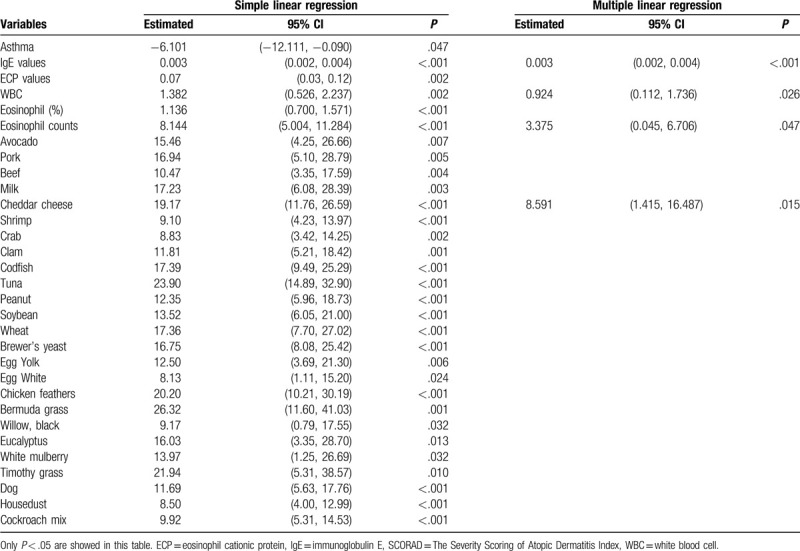
Linear regression for modeling the relationship of SCORAD index and covariates.

The significant associations of Cheddar cheese with other covariates through Phi coefficient analysis were analyzed. The top 3 associations with Cheddar cheese are tuna (*r* = 0.632), codfish (*r* = 0.613), and clam (*r* = 0.613), all of which fall within the seafood classification. Crab and shrimp also have a significantly moderate correlation with Cheddar cheese, with *r* = 0.458 and 0.429, respectively.

## 4 Discussion

In this study, we found that 4 important covariates, serum IgE values, total WBC counts, eosinophil, and Cheddar cheese sensitization, influenced SCORAD index, which increased along with IgE values and specific IgE to Cheddar cheese. Furthermore, some covariates, particularly the seafood (the mainly food sensitization in Taiwan), strongly correlated with Cheddar cheese, which may be caused by cross-reactivity but still need further studies to confirm this finding. The limitations of this study included the missing data of feeding pattern and MAST allergen test, which was not followed with a food challenge test.

Different allergens had various effects on SCORAD index, with cheese on the SCORAD index and sleep disturbance, wheat on the distribution area, and tuna on severity. IgE levels showed a greater correlation with SCORAD index than ECP in childhood AD, thus indicating that the pathogenesis of childhood AD may differ from that of adulthood AD. The findings of this study maybe advise patients who show sensitization to foods to avoid when poor control of AD severity or arrange food challenge test.

In our previous cohort study, we observed egg white sensitization as the key factor in persistent infantile AD.^[17]^ This current work is the first to report an association between Cheddar cheese and childhood AD. Cow's milk is a common allergen, and cow milk-derived cheese retains an appreciable level of allergenicity.^[18]^ However, during the cheese-making process, milk proteins are degraded by chymosin, as well as milk-derived and bacterial proteases. In recent decades, the incidence of food allergy has increased in developed countries. Western diets are high in advanced glycation end-products (AGEs), which come from cooked meat, oils, and cheese. Yao et al previously reported that sensitization to the following allergens indicated a significant association with a higher fraction of exhaled nitric oxide (FeNO) in a Taiwanese population: Dermatophagoides pteronyssinus, Dermatophagoides farina, Blomia tropicalis, cat, German cockroach, oriental cockroach, codfish, crab, shrimp, and cheese.^[19]^ This finding suggests that sensitization to certain food allergens may contribute to rapid airway inflammation.

In conclusion, SCORAD index showed significant association with serum IgE levels but not with ECP levels. The positive laboratory tests of different allergens had varying impact on SCORAD index, sleep disturbance, area, and severity. This study is the first to show the importance of cheese's influence on AD.

## Author contributions

Ho-Chang Kuo acquired and analyzed the data, drafted the article, revised the article for intellectual content, and provided final approval of the version to be published. Chi-Hsiang Chu and Yu-Jih Su acquired data, helped review the article, and provided approval of the version to be published. Chih-Hung Lee helped review the article and provided approval of the version to be published. Ho-Chang Kuo carried out conception and design, acquired the data, revised the article for intellectual content, and provided approval of the version to be published.
